# Editorial: Allergies in focus, tales from the less explored world: prevalence, allergens, and treatment strategies in Latin America and Africa

**DOI:** 10.3389/falgy.2026.1830021

**Published:** 2026-04-07

**Authors:** Renata Curciarello, Cecilia I. Muglia, Paola L. Smaldini

**Affiliations:** Instituto de Estudios Inmunológicos y Fisiopatolológicos, CONICET-Facultad de Ciencias Exactas, UNLP, La Plata, Argentina

**Keywords:** allergy model, global health inequities, Global South, Latin America and Africa, molecular allergy diagnostics

Allergic diseases represent a growing global health burden, affecting hundreds of millions of individuals worldwide. Several evidences have demonstrated that Western environment (or modern urbanization) plays a significant role in the development of these conditions, basically due to the impact of lifestyle on the human microbiome (1). While allergic conditions are higher in developed resourced countries and lower in low resourced countries, they remain to be a growing public health problem, globally (2). However, what we know so far about their increasing prevalence, epidemiology, molecular drivers, and therapeutic strategies, arises mostly from European, North-American or Chinese cohort-studies, whereas allergic conditions remain disproportionately uncharacterized in low-income regions (3, 4). Latin America and Africa—regions marked by vast environmental diversity, heterogeneous genetic backgrounds, rapid urbanization, and profound socio-economic contrasts—continue to be underrepresented in the global allergy literature. This imbalance limits our understanding of region-specific allergen exposures and immune responses and constrains the development of context-adapted diagnostic and management strategies.

This Research Topic was conceived to provide an attractive opportunity to gather reports, scientific research and observational studies addressing prevalence, allergen characterization, and clinical management of allergic diseases within Latin American and African contexts. Although the number of published contributions is modest, the articles assembled here offer meaningful insights into allergic diseases across diverse settings and methodological approaches.

Only one contribution has been proposed by a Latin American group, underscoring ongoing structural and funding constraints affecting research systems in several countries in the region. Restricted access to research infrastructure, fluctuating funding schemes, and broader socio-economic instability continue to limit the capacity to generate and disseminate scientific knowledge. This underrepresentation should not be interpreted as a lack of expertise in the subject or scientific relevance, but rather as an expression of systemic inequities shaping global research landscape. Strengthening regional funding mechanisms and fostering collaborative South–South and North–South networks remain essential to enhance the visibility and sustainability of allergy research from Latin America.

Nevertheless, recent evidence from the region illustrates the scientific relevance of locally generated data. A molecular sensitization study conducted in two Argentine cities with distinct climatic profiles demonstrated marked differences in IgE sensitization patterns to mites, pollens, and fungal allergens, highlighting the profound impact of local environmental conditions on allergic phenotypes. Such findings reinforce the central premise of this Research Topic: environmental diversity and regional context matter, and diagnostic and management strategies must be tailored accordingly (5).

The articles from Africa—two from Tunisia and one from South Africa—highlight scientific vitality emerging from distinct regional contexts and illustrate the diversity of allergic challenges across the continent.

At the experimental level, Andrade-Oliveira et al., developed a standardized murine model of peanut allergy in BALB/c and C57BL/6 mice, representing an important methodological advance. By applying a previously validated food allergy protocol to peanut protein sensitization, the study enables direct comparisons across different food allergens, facilitating mechanistic investigations and improving reproducibility. The identification of strain-dependent immunological responses, including differences in secretory IgA production and intestinal immune parameters, reinforces the importance of host factors in shaping allergic outcomes. Standardized experimental platforms like this are critical for advancing translational research and bridging mechanistic insights with clinical observations.

From an environmental and epidemiological perspective, the South African pilot study conducted in the North-West province of Potchefstroom integrates aerobiological monitoring of airborne allergens with clinical sensitization profiles Gharbi et al. The high prevalence of positive skin prick test reactions and the significant correlations between specific pollen concentrations and allergic rhinitis symptoms underscore the dynamic interaction between local vegetation patterns and respiratory health. The identification of dominant grass and tree pollen allergens, alongside fungal spores such as *Alternaria*, emphasizes the need for region-specific diagnostic panels and accessible testing strategies. The study also highlights gender differences in sensitization patterns and underscores the importance of use affordable, locally adapted diagnostic tools.

The two Tunisian studies focus on the characterization of food allergies using molecular diagnostics, illustrating the expanding role of component-resolved diagnostics in North African clinical practice. The pilot study by Krir et al. about shrimp allergy, characterizes the clinical spectrum of disease and explores cross-reactivity with house dust mites and snails, emphasizing the central role of tropomyosin as a shared immunodominant and cross-reactive antigen. By integrating total IgE, specific IgE, and recombinant allergen measurements, the study provides insight into cross-sensitization patterns in a region where dietary habits and environmental exposures are rapidly evolving, further underscoring the need for regional specific studies.

Complementing this work, the retrospective analysis of IgE-mediated cow's milk protein allergy in Tunisia by Ouerdani et al. demonstrates the prognostic value of molecular allergen testing. The identification of specific IgE thresholds to whole milk and casein associated with disease persistence, offers clinically relevant tools for risk stratification and personalized management. In healthcare settings where resources may be constrained, the ability to predict disease persistence and tailor follow-up strategies represents a meaningful contribution to patient care and public health systems.

Collectively, the articles in this Research Topic reflect the multifaceted nature of allergy research in underrepresented regions, that necessarily involves experimental model development, environmental exposure assessment, molecular allergen profiling, and prognostic biomarker identification. They underscore shared priorities, including the need for standardized methodologies, locally adapted diagnostic strategies, and the integration of molecular tools into routine clinical practice.

The limited number of submissions—particularly from Latin America—should be considered not as a conclusion but as a call to action. Building sustainable research ecosystems requires stable funding, infrastructure investment, training opportunities, and strong international collaboration networks. Allergy research in the Global South is essential not only for addressing local health needs but also for enriching global understanding of how environmental diversity, dietary transitions, infection patterns, and climate change influence allergic disease.

On behalf of the Topic Editors, we thank all authors and reviewers who contributed to this Research Topic and hope that it will encourage further research and collaboration across Latin America and Africa. Addressing inequities in scientific production is fundamental to achieving more equitable health outcomes in a world context where the prevalence of allergic diseases continues to increase.
